# Characterized Bioelectric Signals by Means of Neural Networks and Wavelets to Remotely Control a Human-Machine Interface

**DOI:** 10.3390/s19081923

**Published:** 2019-04-24

**Authors:** David Tinoco Varela, Fernando Gudiño Peñaloza, Carolina Jeanette Villaseñor Rodelas

**Affiliations:** 1 Department of Engineering, ITSE, FESC, UNAM, Cuautitlán Izcalli 54714, Edo. de Mex, Mexico; fernando.gudino@comunidad.unam.mx; 2 Department of Engineering, Technology Bachelor’s Degree, FESC, UNAM, Cuautitlán Izcalli 54714, Edo. de Mex, Mexico; caro408@comunidad.unam.mx

**Keywords:** nano systems, command and control systems, human machine interfaces, integrated circuit interconnections, intelligent control, biosignals, wavelets

## Abstract

Everyday, people interact with different types of human machine interfaces, and the use of them is increasing, thus, it is necessary to design interfaces which are capable of responding in an intelligent, natural, inexpensive, and accessible way, regardless of social, cultural, economic, or physical features of a user. In this sense, it has been sought out the development of small interfaces to avoid any type of user annoyance. In this paper, bioelectric signals have been analyzed and characterized in order to propose a more natural human-machine interaction system. The proposed scheme is controlled by electromyographic signals that a person can create through arm movements. Such arm signals have been analyzed and characterized by a back-propagation neural network, and by a wavelet analysis, in this way control commands were obtained from such arm electromyographic signals. The developed interface, uses Extensible Messaging and Presence Protocol (XMPP) to send control commands remotely. In the experiment, it manipulated a vehicle that was approximately 52 km away from the user, with which it can be showed that a characterized electromyographic signal can be sufficient for controlling embedded devices such as a Raspberri Pi, and in this way we can use the neural network and the wavelet analysis to generate control words which can be used inside the Internet of Things too. A Tiva-C board has been used to acquire data instead of more popular development boards, with an adequate response. One of the most important aspects related to the proposed interface is that it can be used by almost anyone, including people with different abilities and even illiterate people. Due to the existence of individual efforts to characterize different types of bioelectric signals, we propose the generation of free access Bioelectric Control Dictionary, to define and consult each characterized biosignal.

## 1. Introduction

Bioelectric signals represent physiological variables that are relevant for the generation of more natural interfaces between a human and a machine. From such variables, we can obtain commands that can control any type of technological device, and so, human-machine interfaces (HMI) can be designed. 

The physiological variables can be a very small voltage, such as that obtained in an electroencephalogram, or voltages of greater magnitude, such as those that are related to an electrocardiogram. The use of signals of this type to control electronical devices, is of substantial social relevance since all people generate signals of such physical magnitudes, regardless of whether they have diseases or are healthy, and whether they have all their capabilities or present different capabilities. 

In this project we have characterized electromyographic signals (EMGs). Neural networks and wavelet analysis are perfect tools for objectives of this type since such schemes can identify and characterize the behavior patterns of each EMG signal. Once these signals have been characterized, it is possible to generate connections via the Internet to control devices such as Raspberry Pi [1]. 

Recently, many efforts have been dedicated to the development of friendly interfaces for generating HMI that can use biometrics such as voice, vision, gestures, and biosignals as input and output channels. Some of such HMIs may involve the use of bodily movements such as hand movements, which require a multimedia device that can capture them [2,3,4]. 

Nevertheless, the biometric signal we are interested, are the biosignals, for example the bioelectrical brain signals or EEG (electroencephalogram), which are very interesting, and they have been used too as control elements. The first experiment that demonstrated the real possibility of use EEG signals as control elements was performed by Vidal [5], who was the director of the Brain-Computer Interface Laboratory at UCLA, and from that moment different interfaces based on such scheme have been created [6,7,8,9]. The analysis of the EEG signals, helped to develop the brain-computer interfaces (BCIs), the BCIs have been used in many applications such as flying robots [10], robotic wheelchairs [11], humanoid robot control [12], playing with robots [13], control robotic arms [14], among other applications. This kind of interfaces can be used too to help people with physical disabilities [15]. 

Despite the utility of signals such as EEG and interfaces such as BCI, this project focuses its attention over the EMG, and their practical applications. EMG signals are very important variables for the control of technological elements, mainly because they can be obtained in a relatively easy and inexpensive way. Via the study and processing of signals of this type, it is possible today to detect emotions [16], make music [17], and even develop smart clothes [18].

EMG signals have enabled the control of various types of devices. An example of the use of EMG as a control command is presented in [19], where the authors realized real-time control of a robotic arm with four degrees of freedom with such signals.

In [20], EMG signals were used to control mobile phones. In this work, the authors discuss that even using simple processing techniques, it is possible to detect small muscle contractions and, therefore, acquire data.

An MP3 player has been controlled via EMG in [21], thereby demonstrating a potential application of such signals.

In 1997, the Neuro-Engineering Laboratory of the NASA Ames Research Center began an advanced research program: the Extension of the Human Senses Initiative (EHS). EHS was an effort to produce the next generation of machine and software interface technologies, which include human communication tools that utilize EMG and EEG. They developed a biological control of simulation ship type 757 [22].

Recently in [23], Khanna developed a neural network that can classify hand gestures. Using Arduino, they obtained information that was related to hand gestures and that information was processed by a neural network. They characterized three hand gestures: closed fist, pointed index finger, and natural rest position. They demonstrated the suitability of the Arduino microcontroller as a data acquisition tool. 

On the other hand, some works related to the use of the Wavelet Transform (WT) to analyze the EMG signals have been presented, for example, [24] and [25] used the Discrete Wavelet Transform (DWT) to extract characteristics from EMG signals.

According to [26], WT is one of the most powerful signal processing tools to analyze EMG signals. In such a study, the authors investigated usefulness of extraction of the EMG features from multiple-level wavelet decomposition of the EMG signal. They used different levels of various mother wavelets to obtain the useful resolution components from the EMG signal.

Authors in [27] say that classifying EMG signal patterns has been the subject of considerable research effort in recent years. In that study, feedforward error backpropagation artificial neural networks (FEBANNs) and wavelet neural networks (WNNs) based classifiers were developed and compared in relation to their accuracy in classification of EMG signals. 

As a practical experiment, we can see [28] where the authors analyzed the EMG signals coming from two different subjects using a novel integration of Artificial Neural Network (ANN) and wavelet. According to the authors, their results show how their methodology allows to obtain good accuracy in classifying the hand postures, and opens the way to more functional hand prostheses. Following this idea, the authors in [29] say that there is a need in better exploiting EMG signals in order to increase the effectiveness of hand prostheses. They individuated five movements for the wrist-hand mobility. Then they designed the basic electronics and software for the acquisition and the analysis of the EMG signals. 

In the present project, various EMG signals that are related to arm movement have been analyzed, processed by neural networks and wavelets, characterized as control words, and used to manipulate a remote vehicle via the Internet. Generating an HMI controlled in a natural way, using a Tiva-C development board.

## 2. Justification

With the growth of the Internet of Things (IoT) and the increasingly large need to generate interconnections via the Internet to monitor, receive, and control various types of technological and electronic elements, it is becoming increasingly important to generate HMIs that are easy to use, natural in their execution, and can control electronic devices and computer elements via the Internet.

A natural approach to generating control commands is to obtain information about the movements or biological characteristics of the human being, and generate from them a Bioelectric Control Dictionary (BCD), that is, to classify and characterize bioelectric signals to obtain control words that trigger a single instruction, and save them into the BCD. 

From another perspective, it is both socially and technologically necessary for every person to have access to the technology, regardless of the level of study, deep knowledge of scientific concepts and, most importantly, anyone can use it no matter if a body member has lost or been injured. This project allows almost anyone to control electronic elements via the Internet since everyone generates bioelectric signals with everyday natural movements. Hence, a user does not need to learn confusing commands or concepts to control an electronic device

## 3. Preliminaries

In this section, preliminary concepts that are related to the proposed project, tools, definitions, and software and hardware aspects will be introduced.

### 3.1. Artificial Neural Networks

An Artificial Neural Network (ANN) is a computational scheme that is based on the way in which the human brain learns. ANNs are helpful for solving technological problems, especially the identification and characterization of patterns. 

**Figure 1:**
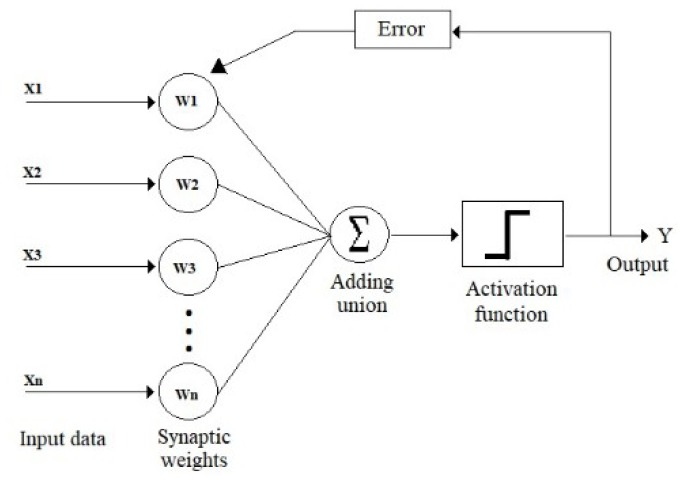
Basic scheme of the perceptron.

**Figure 2:**
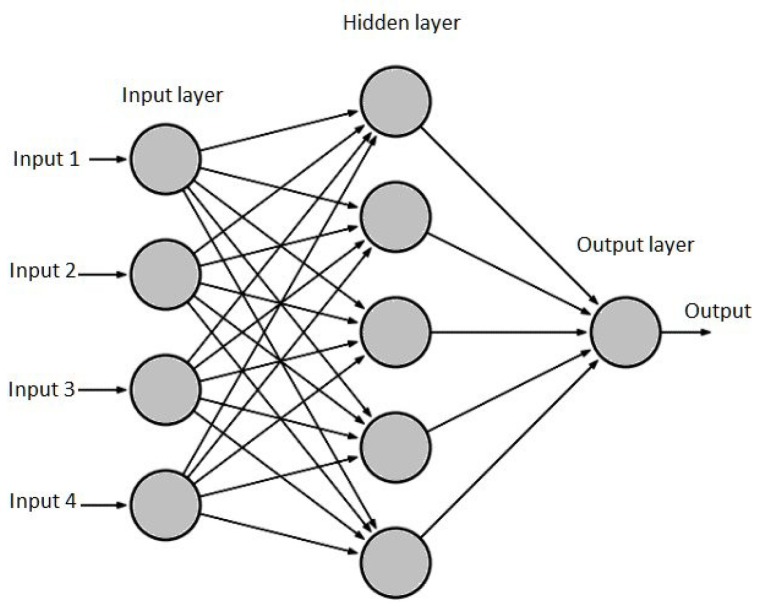
Multi-layer perceptron.

ANNs are structures that are formed by the interconnection of simple neurons. Figure 1 illustrates a simple neuron. Neurons of this type can solve only linearly separable problems. To identify more complex patterns, it is necessary to generate multi-layer networks, such as the showed in Figure 2.

This computational scheme can generalize different behavior patterns based on various groups of data, hence, it has been selected for the characterization of bioelectric signals.

### 3.2. Wavelets

The non-stationary nature of EMG signals makes it difficult to characterize them when using transforms in the frequency domain, such as the Fourier transform. To solve this problem, wavelet transform analysis (WTA) has been used. The WTA provides time and frequency representation of a signal. There are two elementary types of wavelet transform: 

1. The Wavelet Continuous Transform (CWT): Express a continuous *x(t)* signal as an expansion of coefficients proportional to the product between the signal *x(t)* and the scaled and displaced version of the mother wavelet *ψ_a,b_*. The CWT has the next form, where *a* is the scale parameter, and b is is the translation parameter:

$$CWT\left(a,b\right)=\int_{-\infty }^{\infty }x\left(t\right)\psi_{a,b}\left(t\right)dt,\;\;\;\;\;\;\;\;a,b\in R$$

$$\psi_{a,b}=\frac{1}{\sqrt{\left|a\right|}}\psi \left(\frac{t-b}{a}\right).$$

2. The Discrete Wavelet Transform (DWT): Choose values that discretize for parameters a, y, b. The values $a=2^{-j}$ y $b=k\times 2^{-j}$ are usually used. The mother wavelet takes the form:

$$\psi_{j,k}\left(t\right)=2^{\frac{j}{2}}\psi \left(2^{j}t-k\right).$$

Thus, to the mother wavelet ψ(t) a scale function Φ(t) is associated, which allows us to approximate any function x(t) with these functions, by means of the expression:

$$x\left(t\right)=\underset{k}{\sum }\underset{j}{\sum }c_{j,k}\Phi \left(t\right)+\underset{k}{\sum }\underset{j}{\sum }d_{j,k}\psi \left(t\right)\;\;\;\;\;\;\;\;\;j,k\in Z,$$

where $c_{j,k}$ are the scale or approximation coefficients, and $d_{j,k}$ represent the wavelet or detail coefficients of the original signal x(t), with respect to Φ(t) and ψ(t).

**Figure 3:**
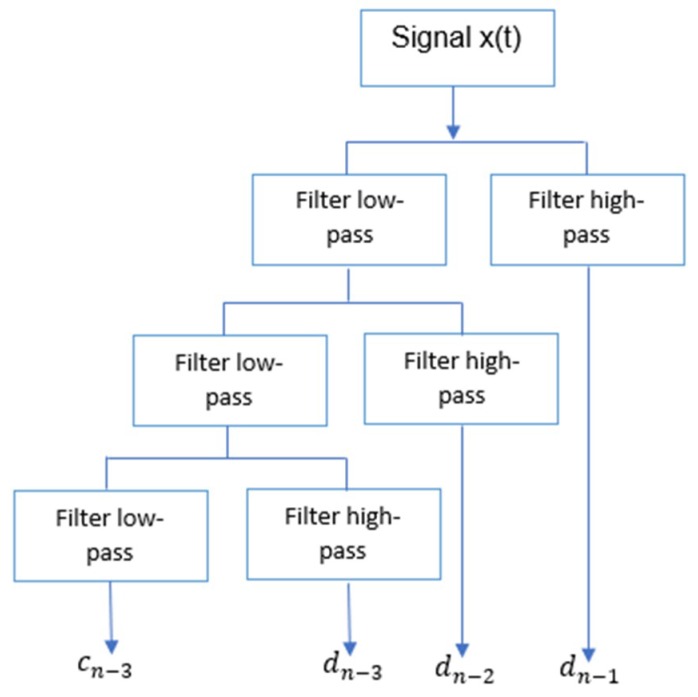
Discrete Wavelet Transform (DWT) decomposition.

The development of algorithms to evaluate the DWT led to the implementation of “filter banks”. These filters correspond to a low-pass filter and a high-pass filter, when the original signal passing through them, the output coefficients $c_{j,k}$ and $d_{j,k}$ respectively, are obtained. A decomposition of the signal can be obtained at different levels by passing the scale coefficients obtained from the previous filtering by an identical pair of filters, thus obtaining the coefficients of the next level, as shown in Figure 3. Such coefficients can describe the EMG signals.

Now, in this project, this scheme has been used to generate control words which can be used to remotely control a robotic vehicle.

### 3.3. Bioelectrical Signals

It is well known that each biological body generates electrical impulses that depend on the activity that is being performed, regardless of whether it is a mental activity or a corporal activity. These electrical potentials are generated by cells that are called excitable cells and their electrochemical activity. All the impulses that are generated by a biological body are called bioelectrical impulses. Depending on the type of activity and the body area that generates them, these signals have a characteristic electrical behavior, as can be seen in Table 1.

These data provide information that depends on the person. Hence, due to the variation of these data, it is possible to obtain medical diagnoses or identify changes that are taking place in the body. In this case, these data are important because we can characterize the information that the human body gives us and, thus, be able to use such information as an element of control.

### 3.4. XMPP

The Extensible Messaging and Presence Protocol (XMPP) is an open protocol that is based on the XML (eXtensible Markup Language), which is designed to provide solutions that are related to real-time communications. 

Some of the most notable advantages of XMPP are: decentralization, safety, extensibility, open standard, and free. Due to its advantages, the XMPP protocol is useful in the development of human-machine interfaces that can control technological devices remotely, such as the interface that is proposed in this paper. 

**Figure 4:**
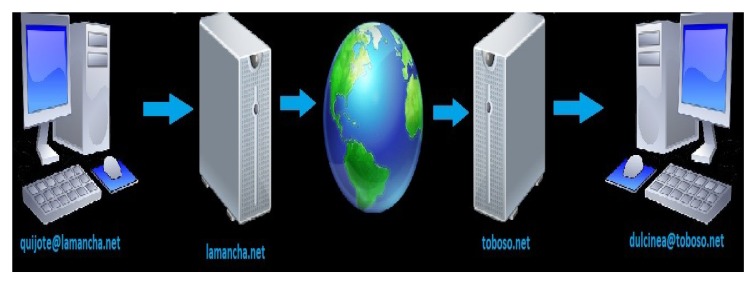
Connection process between two users via the Extensible Messaging and Presence Protocol (XMPP).

Each XMPP entity requires a Jabber identifier (JID), which contains the username and the domain in which the resource is connected. JID uses a format that is similar to an email address “user @ domain/resource”. The message delivery process can be seen in Figure 4.

## 4. Methodology

This section describes the software and hardware that were used to develop this project and how the project was implemented.

### 4.1. Project Description

**Figure 5:**
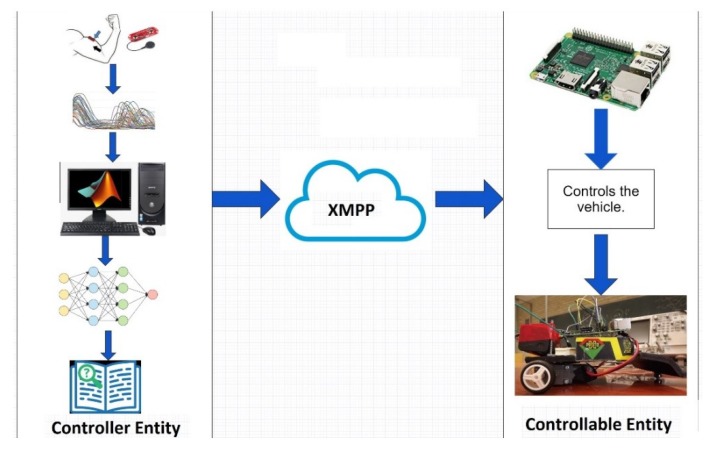
Basic scheme of the proposed project. It is composed of the controller entity and the controllable entity.

A prototype that managed the data acquisition, characterization, transmission, and control was developed; it is illustrated in Figure 5.

**Figure 6:**
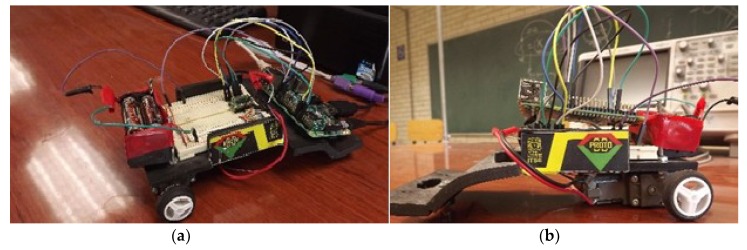
Prototype of the controllable entity, (**a**) seen from above, (**b**) seen from the side.

As shown in Figure 5, the project has two parts: a control entity, and a controllable entity. The control entity is composed of a muscle sensor, a Tiva-C development board, and a computer with the following characteristics: Intel Core i5-3330 CPU, running at 3 GHz, 8 GB RAM memory, and a 64-bit operating system (OS). The controllable entity (Figure 6) consists of a Raspberry Pi 3 development board and a prototype of the vehicle to be managed. The controllable entity is very simple because it is just a Raspberry Pi board that controls the motors of a pair of wheels. The motors can be turned on, turned off, and made to go forward and backward. 

Tiva-C has been used due to the ease of working with the Myoware muscle sensor, the connection between these two devices is direct and does not require complicated or expensive configurations. In the first, it was considered to use the Arduino UNO board to obtain the bioelectrical signals, however, Arduino UNO and Tiva-C can be used in the same way, and can be connected in the same form to the sensor, but Tiva-C can speeds up to 80 MHz, faster than 16 MHz of Arduino. To the authors’ knowledge, Tiva-C has not been tested in this kind of schemes, which was the first reason Tiva-C LaunchPad was selected.

By generating a basic communication script, it is possible to obtain the sensor information directly from Tiva-C and process the obtained data in MATLAB on a computer. These collected data are processed, analyzed and characterized through an ANN, which is developed in MATLAB’s neural network toolbox. Another analysis of such data was made by means of the wavelet concept in order to generate the same characterized data as ANN, wavelet analysis made via MATLAB’s wavelet toolbox.

Once the data that were obtained through Tiva-C have been processed and analyzed, three control words that are related to the characterized arm movements are saved in the BCD. Then, the PC is ready to send the control commands to the controllable entity.

The controllable entity has the Raspberry Pi 3 development board as its processing core. This board has been selected because it has a Wi-Fi connection and it has been tested and analyzed for use in the IoT [30].

The Raspberry board has been loaded with the Raspbian OS. The software that is responsible for receiving and processing the control words from the control entity and managing the outputs of the development board has been developed in the Python programming language.

When the controllable entity connects to the XMPP jabberes.org server, it can begin to receive information from the control entity. In this case, depending on the instruction that is received remotely, the controllable entity will manage the GPIOs and the output actuators.

For the connection of the control entity and the controllable entity, it was not necessary to use our own servers or clouds since the XMPP communication protocol has its own servers, which are free to access.

### 4.2. Obtaining the Signals

**Figure 7:**
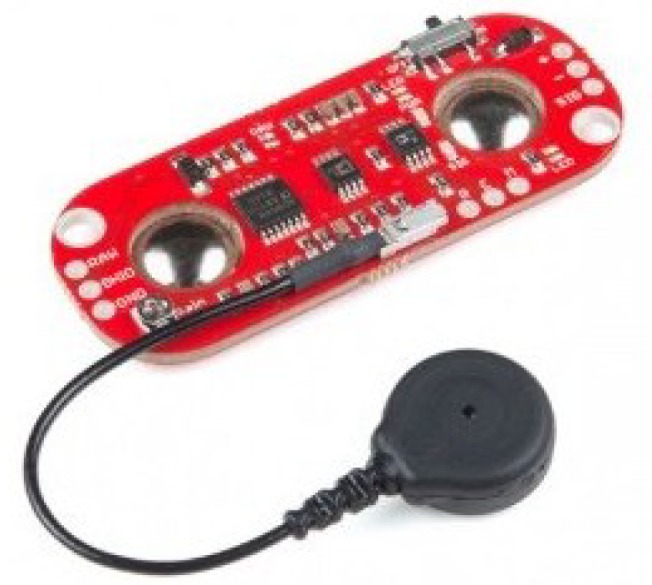
Myoware muscle sensor.

**Figure 8:**
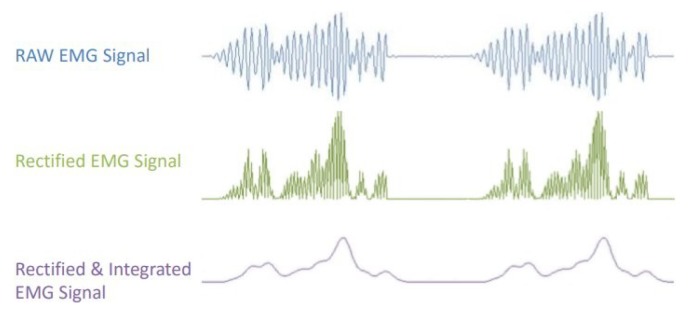
Image that was obtained directly from the Myoware muscle sensor’s datasheet. The obtained electromyographic signals (EMGs) signal is processed before its output.

To obtain bodily bioelectrical signals, it was necessary to use a muscular sensor. In this case, the Myoware Muscle Sensor, which is shown in Figure 7. This sensor has the advantage that it does not provide a raw signal; instead, it processes the EMG signal before providing it to us. It processes, filters, rectifies, and amplifies bioelectric signals, as shown in Figure 8, which is obtained from its datasheet [31]. The correct form for connecting the sensor to a muscle is provided in [31].

The sensor obtains the EMG signals from the human body and sends the information to the Tiva-C board. At this moment, the behavior of the signal can be observed via the serial plotter function. It is easy to obtain and interpret such data. The acquired data are analyzed and characterized in the next step of the system.

The first bioelectric signals that were studied and characterized were signals that correspond to three compound movements of the arm. To make these characterizations, each person who participated in the experiment was placed in a straight sitting position, with his elbow resting on a desk. In that position, three types of arm movements were made: the arm upward movement, a double upward movement, and an upward-downward movement. We obtained 200 samples of each movement and each one was saved as a vector with 150 sampling points. 

The 200 vectors that were obtained from each movement were used to train the supervised ANN, and they were analyzed by means of wavelets. In this way, three characterized control words that are used in our experiments have been obtained. 

**Figure 9:**
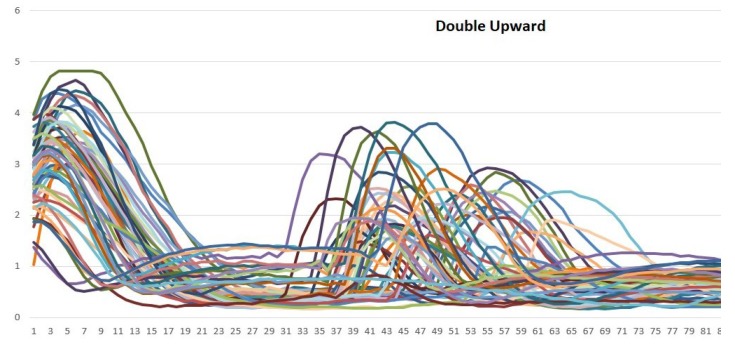
Samples that are related to the double upward movement. The *Y*-axis represents each sampling point of the vector that is related to each sample and the *X*-axis represents the signal amplitude (it was mapped between 0 and 5).

**Figure 10:**
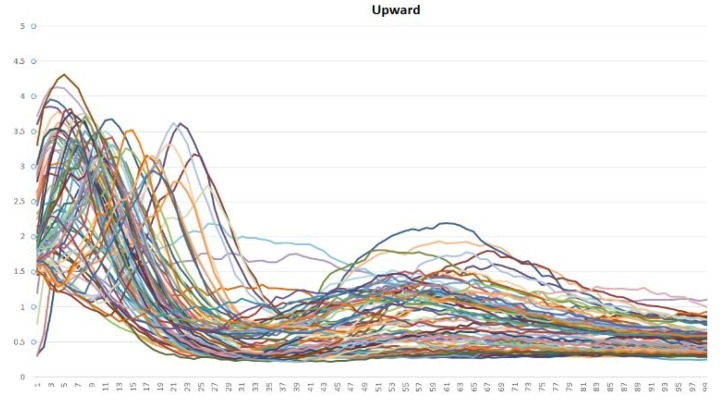
Samples that are related to the upward movement. The *Y*-axis represents each sampling point of the vector that is related to each sample and the *X*-axis represents the signal amplitude (it was mapped between 0 and 5).

**Figure 11:**
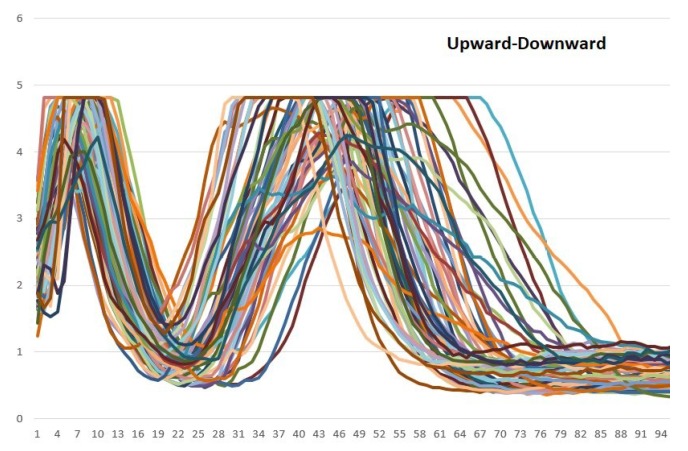
Samples that are related to the upward-downward movement. The *Y*-axis represents each sampling point of the vector that is related to each sample and the *X*-axis represents the signal amplitude (it was mapped between 0 and 5).

According to Figure 9, Figure 10 and Figure 11, different bioelectrical signals are obtained from the arm movements. This research has focused on analyzing in depth the patterns in the EMG signals that are related to the described movements.

### 4.3. Signal Processing via Neural Networks

After the compound arm movements were clearly identified and the signals that correspond to those contractions were obtained, it was necessary to interpret the analog signals that were collected from the muscle sensor. For the interpretation and analysis, an interface was developed in MATLAB that graphs the data that are obtained by the sensor in real time.

The design of the interface is described as follows:

1. Connection of the muscle sensor with the Tiva-C and MATLAB: The output of the muscle sensor is interpreted with the analogue-digital converter of the corresponding board. With such data, a program was designed that interprets the acquired data through the sensor and converts the database to a digital code, which is sent to MATLAB. After that, a script was designed in MATLAB to save the information that is related to the arm movements. It obtains the data that are sent by the microprocessor board and stores such data in a vector; each saved vector has the following form:
  Vsignal {150} = {samplingpoint0, samplingpoint1, …, samplingpoint149}
2. Signal graph in MATLAB: With the data that have already been stored, the obtained signals can be plotted. For the experiments, 200 samples were considered, which vary between 0 and 5 V (the original values were mapped to be in accordance with the board values). In this way, a graphic generator was obtained. It enabled the monitoring of the muscle behavior in real time.
3. Analysis of patterns: Using the previously designed tool, the behavior patterns of the EMG signals from the arm movements were analyzed. According to Figure 9, Figure 10 and Figure 11, each movement has a unique form. Many samples are shown in the plots and although there are small differences among them, they share a general and distinguishable behavior; hence, they have very similar patterns of behavior.
4. Training of the neural network for the recognition of patterns: Once the wave signals were obtained, the next step was to prepare and train a neural network for the automatic recognition of the bioelectric patterns.
  For this, a neural network was designed and implemented in the MATLAB’s neural network toolbox, which will enable us to obtain different samples of EMG signals and classify them according to the type of signal to which they correspond. Then, it is necessary to perform supervised training of the back-propagation neuronal network.
  Once the ANN has been trained, 3 control words are generated and saved into the BCD. These calculated weights are used to evaluate every movement that a user performs.

### 4.4. Characterization via Wavelets

As was said before, another analysis was made over the 200 vectors that were obtained from each arm movement, this analysis was made via wavelets, the idea of this process is to obtain the same control words that were obtained through neural networks and control the vehicle via XMPP.

The vectors shown in Figure 9, Figure 10 and Figure 11, were analyzed in this way. In order to choice the mother wavelet, two criteria were implemented, the first was based on finding a wavelet that had a similarity with the signals to be analyzed, and the second was to make different tests with the wavelets selected from the previous step, choosing the one that presented a better approximation with the signals to study.

**Figure 12:**
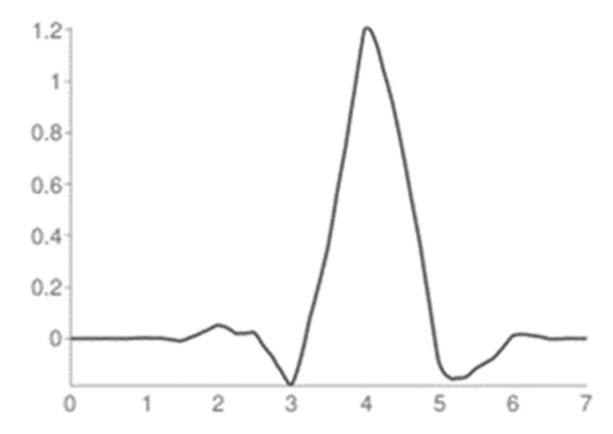
Wavelet Symlets 4 (sym4).

Through MATLAB’s Wavelet toolbox, the analysis of our signals was performed. This toolbox allows us to select, at the beginning, the type of transformation that we want to do, in this case the DWT, the mother wavelet with which we want to analyze the signal and the level of decomposition that we want to reach. For the analysis of our signals, Wavelet Symlets 4, presented in Figure 12, was implemented as the mother wavelet; In addition, 3 decomposition level was chosen.

**Figure 13:**
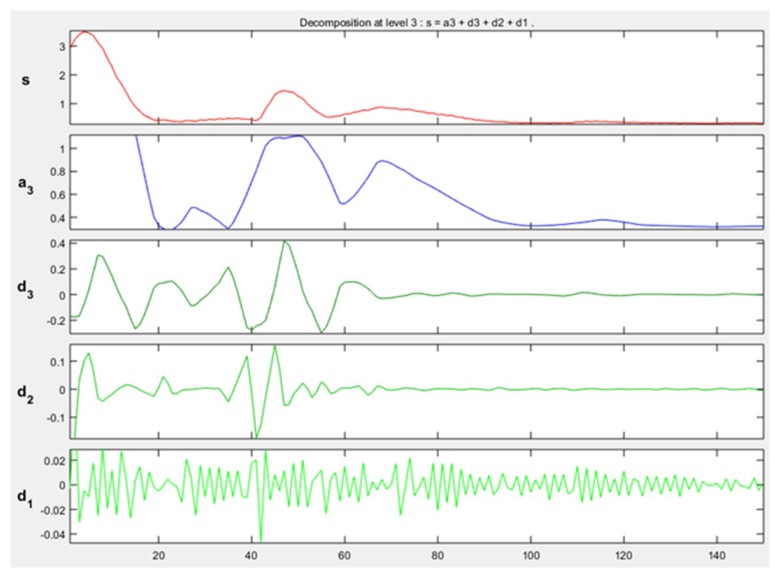
Example of approximation and detail coefficients, approximation to level 3 related to one double upward movement.

**Figure 14:**
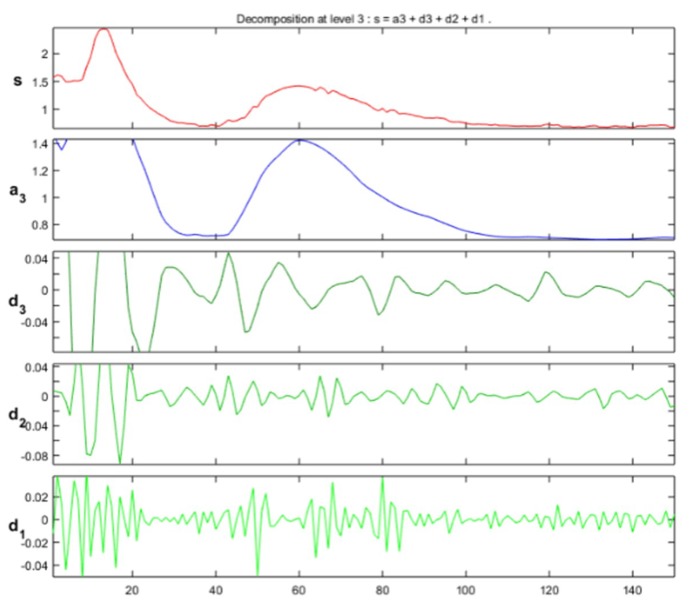
Example of approximation and detail coefficients, approximation to level 3 related to one upward movement.

**Figure 15:**
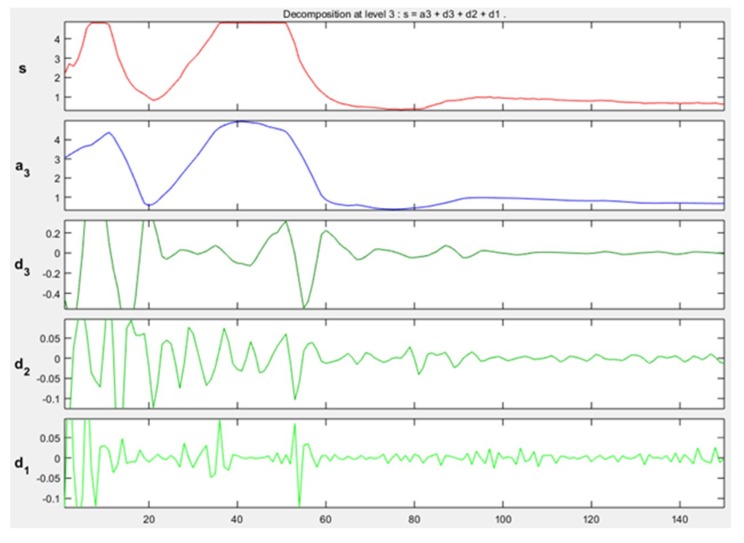
Example of approximation and detail coefficients, approximation to level 3 related to one upward-downward movement.

The approximation and detail coefficients were obtained for three approximation levels, which are presented in Figure 13, Figure 14 and Figure 15, for the movements double upward, upward, and upward-downward, respectively, where *s* represents the signal to study, $a_{3}$ is the approximation to level 3 and $d_{1},\;d_{2},\;and\;d_{3}$ are the details at level 1, 2, and 3.

**Figure 16:**
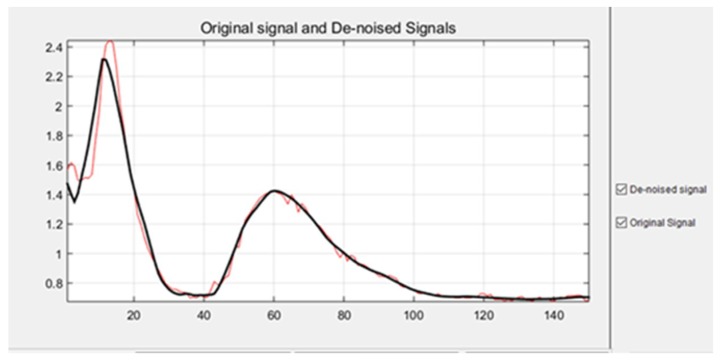
Comparison between a signal with noise, and a de-noised signal. In this case, the signal corresponds to a sample from the upward movement.

In order to analyze the complete behavior related to each described arm movement, it was necessary to obtain all the characteristics from each of them. This means, all the vectors related to each of the arm movements were analyzed. First of all, it was necessary to remove all the noise from the original signals, in Figure 16, it is possible to see a comparison between an original signal and the de-noised signal.

**Figure 17:**
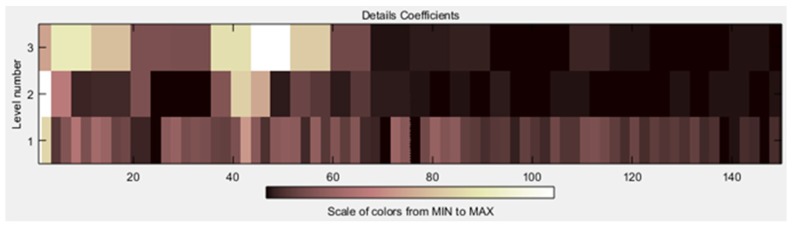
Detail coefficients related to the double upward movement.

**Figure 18:**
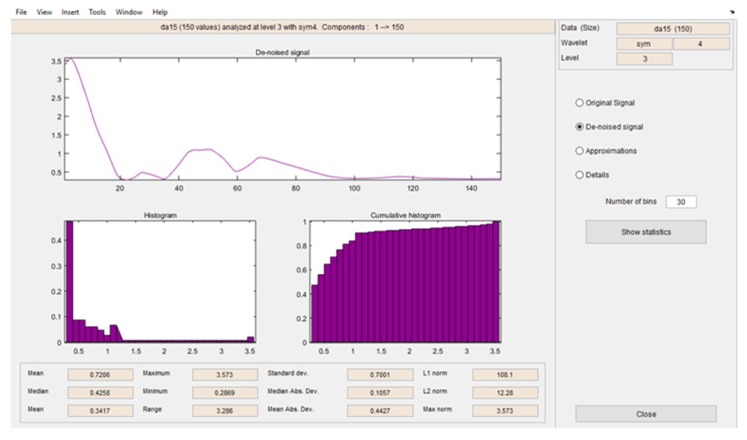
Statistical data of the de-noise signal of the approximation coefficients in level 3 related to the double upward movement.

After the signals were de-noised, it was necessary to obtain all the characteristics related to the signal. In Figure 17 and Figure 18, it is possible to see the detail coefficients and the statistical data of the de-noise signal of the approximation coefficients in level 3 related to the double upward movement.

**Figure 19:**
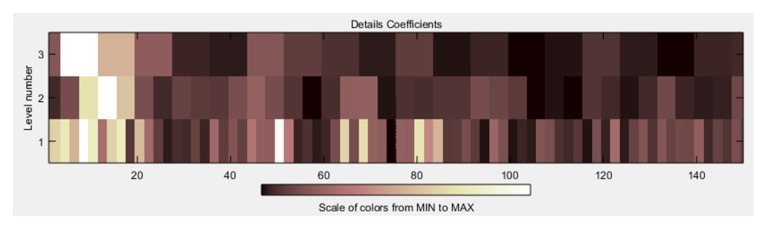
Detail coefficients related to the upward movement.

**Figure 20:**
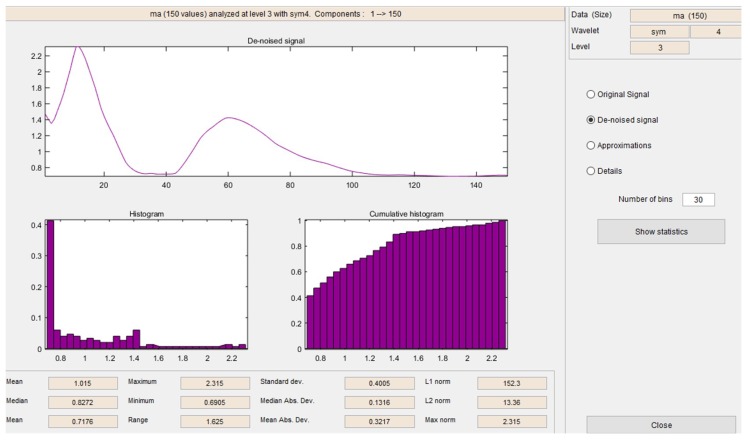
Statistical data of the de-noise signal of the approximation coefficients in level 3 related to the upward movement.

In Figure 19 and Figure 20, it is possible to see the detail coefficients and the statistical data of the de-noise signal of the approximation coefficients in level 3 related to the upward movement.

**Figure 21:**
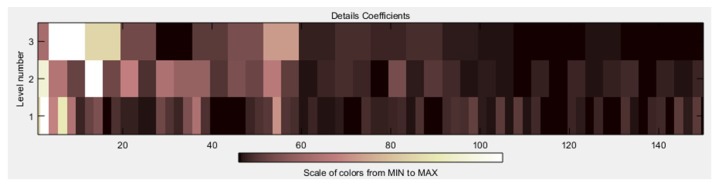
Detail coefficients related to the upward-downward movement.

**Figure 22:**
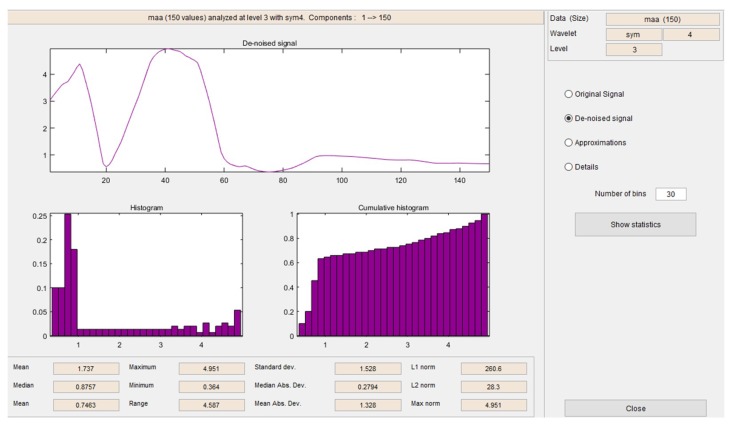
Statistical data of the de-noise signal of the approximation coefficients in level 3 related to the upward-downward movement.

In Figure 21 and Figure 22, it is possible to see the detail coefficients and the statistical data of the de-noise signal of the approximation coefficients in level 3 related to the upward-downward movement.

The approximation coefficients of level 3 were taken as the characteristic data vector for each of the EMG signals, to which a statistical description was made, obtaining statistical data such as the maximum value, the minimum value, the average, the sum, among others. After performing this analysis, it was calculated an arithmetic average, in which all the signal coefficients are used. In this case it is necessary to say that the signal coefficients were calculated for each vector related to each arm movement. One interesting thing related to this fact, is that the average value related to the coefficients of each signal has a particular value for each of the arm movements. Some examples of some vectors related to this behavior is shown in Table 2. In Table 2, are shown 5 of 200 samples from each arm movement, and it is possible to see, that each average value related to the three arm movements have a similar value.

With the obtained coefficients values, it is possible to see that there is a general behavior related to each arm movement, indeed some coefficients were proved in a raw form, to generate control words, however, we have used such coefficients as input data in a classification tool, a neural network.

The training patterns of the neural network that are obtained from the wavelets have the shape shown in the Figure 13, Figure 14 and Figure 15, represented with the coefficients showed in Figure 17, Figure 19 and Figure 21. As showed in Figure 16, the wavelet analysis compared with a direct signal from the sensor, reduces the intrinsic noise level of the EMG signal, thus the level of classification error is reduced.

### 4.5. Controlling a Vehicle via the Internet

**Figure 23:**
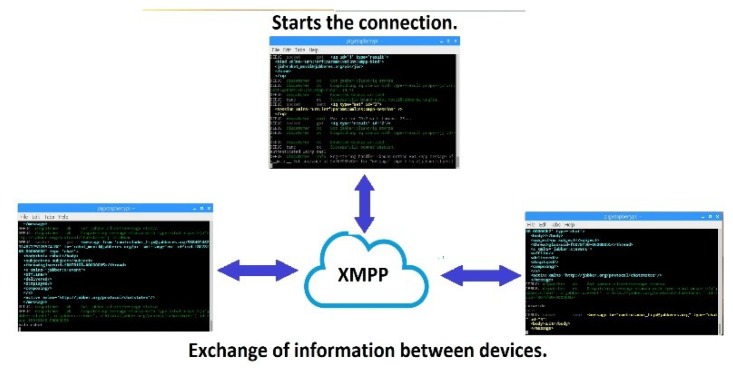
The connection via the Internet. First, it is necessary to establish the connection between the devices. After that, the devices will communicate with each other.

With the trained ANN and the data obtained from the wavelet analysis, a user can begin to send control words to the controllable entity via XMPP. Figure 23 illustrates this transmission behavior, in which it is possible to observe the connection via the Internet.

As shown in Figure 23, when a user makes an arm movement, it sends the correct control word via the Internet using the XMPP protocol. In this manner, the control word can travel through the world. Because of this protocol, the proposed system can use any server that is installed in any geographical position, without restrictions. The controllable entity receives the control word and processes the information to manipulate the electronic device remotely. 

In this case, the response is very simple: the unique action that the controllable entity must perform is to recognize the received command and manage the corresponding Raspberry’s GPIO. The BCD was previously saved to the Raspberry board.

## 5. Results

The muscular bioelectrical impulses that were generated by the compound arm movements have been studied and characterized by mean of ANN and wavelet analysis.

A third level wavelet analysis was used to characterize the movements used as control words. At this level of decomposition, three output scales are produced and averaged for the selection of control variables used subsequently as shown in Table 2. We can see that the coefficients of each movement have a useful central value for their use as makers of control words.

Three muscular movements have been characterized and their corresponding bioelectric reactions have been obtained. Hence, three control words by two different methods have been generated and saved in the BCD. Additional movements were characterized, however, we focused on the three specified movements because they were the most stable. We wanted to characterize the movement of “twist the arm”, however, this case was very difficult because the vectors differed substantially among tests. Hence, it was not possible for us to characterize this movement.

A back-propagation neural network was generated for the bioelectric characterization. This neural network was constructed using MATLAB’s neural network toolbox. Additionally, a wavelet analysis was made in order to obtain the same control words as the neural network characterization, this analysis was executed using MATLAB’s Wavelet toolbox.

A human-machine interface was developed. It directs remotely a vehicle by controlling its direction (forward, backward) and turning it off and on via the characterized electromyograms. The core of the vehicle is the Raspberry Pi, for that reason EMG signals can be used in the IoT network as active elements.

The biosignals have been acquired by means of the Tiva-C LaunchPad board. In this sense, it has been proved that this development board is useful in the design of EMG-based HMI. To the authors’ knowledge, it has not been exploited in these kind of scheme. The HMI has been implemented on two technological platforms, namely, Raspberry and Tiva-C and it is possible to implement it on other similar platforms that are cheap and easily accessible.

The process of characterizing additional bodily electrical responses has begun and is expected to generate additional control words to be entered into the BCD. 

The proposed prototype has been probed. A vehicle that was 52 km away from the control entity was controlled. Three words in our BCD were generated, which were related to turning the vehicle on/off, making the vehicle go forward, and making the vehicle go backwards.

The controllable entity was remotely controlled successfully via EMG signals.

### 5.1. Comparison

There are several HMIs that use the EMG signals as a starting point to generate different control words or characterizations, in [32], it is possible to review advances related to this type of interfaces, they used classification tools, and the classified signals. However, for the analysis of our proposal, we will compare it with respect to some previous EMG-based schemes [23,33,34,35,36], and some BCI schemes [13,15,37].

Table 3 presents the comparative data, where we have that each column represents: the reference from which such data comes, the used device for the acquisition of data and its approximate cost in USD, the used classification tool, the classified movements and the place where the sensor was placed, and finally, the mode of communication, its coverage, and its application.

As we can see in Table 3, the given proposal is the most economical at the hardware level, which is a great advantage for its implementation in different population sectors. And an important aspect that we can observe, is that our proposal is the only one that has a global coverage, making it appropriate for new communication paradigms, such as the IoT.

If the proposed scheme is compared with the BCI schemes in Table 3, it is possible to see that it has some advantages over them, according to [15], the EEG acquisition equipment with high precision is expensive and inconvenient to wear, and the proposed scheme can be easily carried because of its small size. On the other hand, according to [37], it is necessary to train to the users to reach the calm condition (in terms of brain activity), in our proposal, this is not necessary, a user just needs to move the arm. Something interesting to observe, according to the articles referenced in Table 3, and some cases mentioned in [32], different movements of the hand, the arm (including the new compound movements studied in this paper), and the fingers have been characterized, that is, each of these individual efforts has given as a result, possible commands from different body parts. For an individual effort, it would be complicated and time consuming to achieve the objective of characterizing all the corporal movements, for that reason, the idea of generating a public database where each movement can be stored and consulted, could allow us to reach the stated goal more quickly. If all these possible control commands could be defined within a Bioelectric Control Dictionary, future HMIs could download the entire dictionary to their memory, and be interactive with the user’s body movements since the first moment. For these reasons, the creation of a public BCD is proposed.

### 5.2. Advantages

The proposed system has several interesting advantages that make it a good option for developing more realistic schemes:

1. It is cheap to implement. According to Table 4, the average cost of hardware implementation is approximately $114 USD. For software, the use of XMPP enables us to generate connections via the Internet for free. The Raspbian OS is also free, however, the neural network and the wavelet analysis have been implemented in MATLAB, which has a cost for its home version of $149 USD (United States Dollars), other low-cost alternative tools can be used for the classifier.
2. It is easy to implement since all the electronic elements and computational tools are easy to obtain and connect. Every electronic device that is used can be found in virtual stores and physical stores without difficulty. For software such as XMPP, only the installation and generation of users via the Internet are necessary.
3. It can be used by practically anyone, regardless of whether they have physical deficiencies or not. This is an extremely important aspect since the standard HMIs typically require cumbersome devices that must be directly manipulated by the hands. In other cases, it is necessary for the user of such an interface to have deep knowledge about its operation, however, almost anyone can access the proposed system because it is not necessary to have prior knowledge of the operation of the device. The human-machine interface is controlled with body natural movements that are performed every day. If a person has suffered the loss of a body part, he may still control the device since he uses the bioelectrical bodily signals that are generated regardless of whether he is healthy or not.
4. A person who has no technical knowledge or previous training, or is even illiterate, can access this system since it is not necessary to learn any details about the system to generate a remote control. The person only needs to perform the natural movements of his body to send the required control word. Everyone knows how to move his body; it is not necessary to teach anyone how to do it.
5. It can be connected to the IoT. Since communications are being generated via the Internet, in this case, communication depends directly on when the user wants to send the control word. In the future, we seek to create an interface that does not wait for the user’s order but is ahead of the needs of the user, such that the communication is generated directly.
6. Although the proposed interface does not occupy a large space, and it can be connected with relative ease to the user, it must be miniaturized. For this purpose, the complete design of this type of interfaces has been imagined by means of nanoelectronic elements and nanosensors, in this way the size of the interface will be reduced, and therefore it can be connected in practically any part of the body, and so, it cannot be seen as a foreign object, which would present an even greater advantage of our system with respect to other types of alternatives. At this point, it is necessary to mention, the size of the proposal is an advantage with respect to BCI, because the sensors used in such schemes are inconvenient to wear.
7. Since this project focuses on being able to interact in the IoT, one of the most important aspects to consider is the cryptographic security of the information. In this case, because the XMPP protocol already has cryptographic protection algorithms, the transmission of data via the Internet is already protected. Hence, we have a safe and reliable system.

## 6. Conclusions

The muscle movements of the human body can be used as control words for electronic devices of any type, since the EMG signals that are generated by the muscles present consistent patterns of behavior, and such patterns can be recognized by classification systems that are based on neural networks and wavelet analysis. 

Three EMG signals related to compound arm movements, have been obtained, characterized, and analyzed in this paper, such movements have not been analyzed previously. The obtained EMG signals were characterized by means of two different schemes: Neural Networks and Wavelet Analysis. For the characterization by means of neural networks, it was essential to execute supervised training for the designed ANN and it was necessary to have as many as 200 samples for each of the movements that were obtained from various subjects. Differences in EMG signal amplitude among subjects were observed, however, the signals exhibit the same general patterns. For the characterization by means of Wavelets, the signals were de-noised, and were analyzed with a 3 decomposition level.

In this paper, the basic functions of a remote-controlled vehicle were managed via the electrical impulses that were generated by arm movements, and the obtained information was processed in Tiva-C, Raspberry, and MATLAB software. The processing, characterization, and use of the bioelectric signals as a way to remotely control devices of various types can be implemented in schemes such as the IoT because we show that it is possible to generate prototypes that react automatically to specified bioelectric impulses and send such information to any device in any geographical position.

The Tiva-C board has been used instead of the most popular boards, in this case, it has been a useful tool to process the EMG signals. In this same sense, it is possible to say that Tiva-C is an efficient board when it is implemented in EMG-based HMI. It is faster than Arduino UNO board (the most used board), but it has basically the same price, and it has not been exploited in schemes like this previously. This board served as a data acquisition system.

The proposed HMI was compared with previous EMG-based schemes, and with some BCI. The proposal is the cheapest scheme, and it has global coverage of communication with respect to the EMG-based schemes. When compared with some BCI, our proposal shows the advantage that a user does not need to be trained to use it, a user just needs to move the arm. It is small, and for that reason convenient to wear.

Due to the proposed HMI uses the XMPP protocol, it can transmit information via Internet in a safe way, because XMPP has its own cryptographic algorithms.

The proposed human-machine interface can be used by almost any person because regardless of whether the user is healthy or suffers from a disease or body part loss, the user can use the interface and control devices remotely because the HMI will interpret the bioelectric impulses that the user sends, such impulses are generated by the body at all times and under all circumstances.

Similarly, a person who lacks specialized technical preparation, or are even illiterate, can access this system because complex knowledge of the interface is not required. It is sufficient to generate the natural movements of the body to control a remote device.

One of the most important aspects to develop interfaces which do not generate inconvenience in a user, is to limit the size of them. For this reason, future work aims to develop the proposed system by means of nano electronics, in such a way that a nano interface is obtained that is useful for any person, and that can be used in the most possible natural way.

According to the mentioned characteristics, we can conclude that the proposed EMG-based HMI has advantages over other similar HMIs due to its price, size, security, and coverage and it can be used in almost any environment.

Additionally, we will seek to generate an open BCD, in which any researcher, or electronic device, can define and obtain control words related to EMG signals. And so, future devices can connect directly to it and load pre-defined control words into its memory. This proposal is made, due to the existence of individual efforts to characterize different types of bioelectric signals, then, it is necessary to define all the characterized biosignals in an open access repository in order to generate more complex interactions between the human body and a natural HMI.

## Figures and Tables

**Table 1:** Characteristics of different bioelectrical signals.

Bioelectrical Type	Amplitude Range	Frequency Range
Cardiac electrical activity	0.5–4 mv	0.01–250 Hz
Brain electrical activity	5–300 μV	DC-150 Hz
Gastric electrical activity	10 Μv–1 mV	DC-1 Hz
Nervous electrical activity	0.01–3 mV	DC-1 kHz
Retina electrical activity	0–900 μV	DC-50 Hz
Muscular electrical activity	0.1–5 mV	DC-10 kHz

**Table 2:** Average values obtained from the approximation coefficients for each of the signals related to each arm movement. In column one, it is possible to see the average value of five samples, or vectors, related to the double upward movement, and so on in every column. In this sense, this behavior is used to generate control words which represent each arm movement.

Double Upward Movement	Upward Movement	Upward-Downward Movement
0.9030	0.9146	1.7375
0.7864	0.9146	1.5771
0.7612	0.9146	1.7366
0.7206	0.9896	1.7366
0.8567	0.9789	1.4033

**Table 3:** Comparison between our proposal and previous EMG-based and brain-computer interface (BCI) schemes.

Reference	Acquisition Device (Approximated Price)	Classification Tool	Hand/Arm Gestures (Sensor Placed)	Communication and Application
[33]	Neurobelt ($ 760)	A single layer artificial neural network with regularized cost function	No specified.	Local, wireless. Mobile robot Boe-bot (just left and right turns were made by EMG).
[34]	Surface sensor Myoband ($ 149, estimated)	Wavelet decomposition.	Hand: Rest, fist, wavein, waveout, fingerspread (forearm).	Local, Bluetooth. No application.
[35]	Nexus -10 with Myoscan-pro EMG sensor ($ 355)	K-nearest neighbor, Bayes classifier, and combinations of both.	Hand: Press, Left, Right, Circling. (forearm)	Local, RC (Radio control). RC car.
[36]	Biopac MP30 ($ 299.99)	Construction of successive feature vectors for each gesture. Classification referred to as the context-dependent classification, which is carried out within the framework of Bayes theorem.	Arm: 12 symbolic drawing in the air gestures. (shoulder and elbow joints)	Local, wired. No application.
[23]	Arduino MEGA and Myoware muscle sensor ($ 60)	Feed forward neural network	Hand (fingers): Hand closed, Point index finger, Natural resting position. (forearm)	Local, wired, No application.
[13]	EMOTIV ($ 299)	Control actions were trained within the EMOTIV environment.	Head: Two movements called cold and hot.	Local, wireless, HTTP protocol. Control of a wheeled robot.
[15]	Neuroscan ($ 635)	Wavelet decomposition	Head: emotion characteristics.	Local, wireless, Radio frequency. Control of a home auxiliary robot.
[37]	NeuroSky MindWave ($ 99.99)	Fourier method	Head, brain activities. One movement, the robot’s speed.	Local, Wireless LAN. Control of Robotino mobile robot.
Ours	Tiva-C, and Myoware muscle sensor ($ 50)	Neural network back propagation algorithm, and Wavelet analysis	Arm: Upward, double upward, upward-downward.	Global: Wi-Fi. Remote vehicle.

**Table 4:** Cost of the implemented hardware.

Device	Price in USD
Raspberry Pi 3	40
Tiva-C	25
Myoware	38
DC motors and pair of wheels	8
H bridge	3
Total amount	114
